# Case Report: Aortoesophageal fistula after radical gastrectomy for cardia cancer: diagnostic problems and successful staged treatment

**DOI:** 10.3389/fcvm.2026.1806417

**Published:** 2026-05-08

**Authors:** Kai Yang, Meng Dai

**Affiliations:** 1 Department of Interventional Radiology, The Fourth Hospital of Hebei Medical University, Shijiazhuang, Hebei, China; 2 Department of Nuclear Medicine, The Fourth Hospital of Hebei Medical University, Shijiazhuang, Hebei, China

**Keywords:** aortoesophageal fistula, gastric cardia cancer, long-term survival, multi-disciplinary collaboration, TEVAR, thoracic endovascular aortic repair

## Abstract

An aortoesophageal fistula (AEF) following radical surgery for gastric cardia cancer is a rare and serious problem with a historically dismal prognosis. Diagnosing it was difficult because the patient did not show the typical set of symptoms and the initial digital subtraction angiography (DSA) failed to reveal the underlying pathology. Treatment involved a planned multi-specialty approach. First, emergency stent placement in the aorta (TEVAR) was performed to achieve hemostasis. Later, planned surgeries were performed including esophageal exclusion and then colonic interposition to rebuild the food passage. This combined method led to the patient surviving for over three years. This case underscores the necessity for clinicians to maintain a high index of suspicion for aorto-esophageal fistula (AEF), even when initial diagnostic evaluations are unremarkable. It also shows that TEVAR alone is often not enough and a carefully planned series of operations to control both bleeding and infection can lead to a favorable long-term outcomes.

## Introduction

An aortoesophageal fistula (AEF) represents a catastrophic and life-threatening communication between the thoracic aorta and the esophagus. It is an exceedingly rare but fatal complication. The reported incidence is ranges from 0.1% to 0.8% among patients undergoing such surgeries. The mortality rate is extremely high, often exceeding 50%–90% for secondary AEFs if not managed emergently (1). Gastric cardia cancer is a specific type of stomach cancer. It exhibits significant similarities to esophageal cancer regarding its pathogenesis and histological appearance. This disease remains a significant global health challenge. Surgery is the primary treatment modality for gastric cardia cancer but associated with potential complications. AEF is one of the most severe complications. The condition typically presents with chest pain, followed by a sentinel hemorrhage, and subsequently fatal exsanguination (2). The problem often originates from a leak at the anastomotic site. Digestive juices collect and cause infection, which damages the nearby aortic wall leading to a fistula (3). Contrast-enhanced computed tomography (CT) angiography is the primary and the most sensitive imaging modality for the detection of this condition, frequently demonstrasting features such as ectopic gas near the aorta, perigraft fluid, or active contrast extravasation (4). We report this case for several key reasons. These reasons highlight its value for teaching and practical application.

Firstly, it illustrates how an initially contained postoperative leak can rapidly progress to a life-threatening AEF. This necessitates vigilant monitoring. Secondly, it highlights diagnostic difficulties. Early AEF symptoms such as chest pain or minor hematemesis may mimic benign conditions, leading to potentially fatal delays in diagnosis (5). In this case, even a modern imaging test (DSA) initially revealed no abnormalities. This discrepancy was likely attributable to temporary occlusion of the fistula. This underscores the necessity for clinicians to perform repeat imaging if they strongly suspect AEF. Advanced imaging like DSA can show a false-negative result at first if a transient thrombus blocks the fistula. Consequently, diagnostic challenges arise, necessitating repeat evaluations when clinical suspicion persists (6). Thirdly, this case demonstrates the necessity for and successful implementation of a complex, staged, and multidisciplinary treatment plan.

The team employed a multimodal approach combining endovascular methods, emergency surgery, and definitive thoracic reconstruction. Such a result stands in stark contrast to the typically poor prognosis associated with AEF and suggests a possible new treatment model the case underscores critical lessons, such as the necessity of avoiding diagnostic errors stemming from reliance on a single negative angiogram. It advocates for a distinct integrated strategy that combines rapid endovascular control with subsequent surgical source control and gastrointestinal reconstruction to achieve a lasting outcome.

## Case presentation

### Patient information

**Figure 1:**
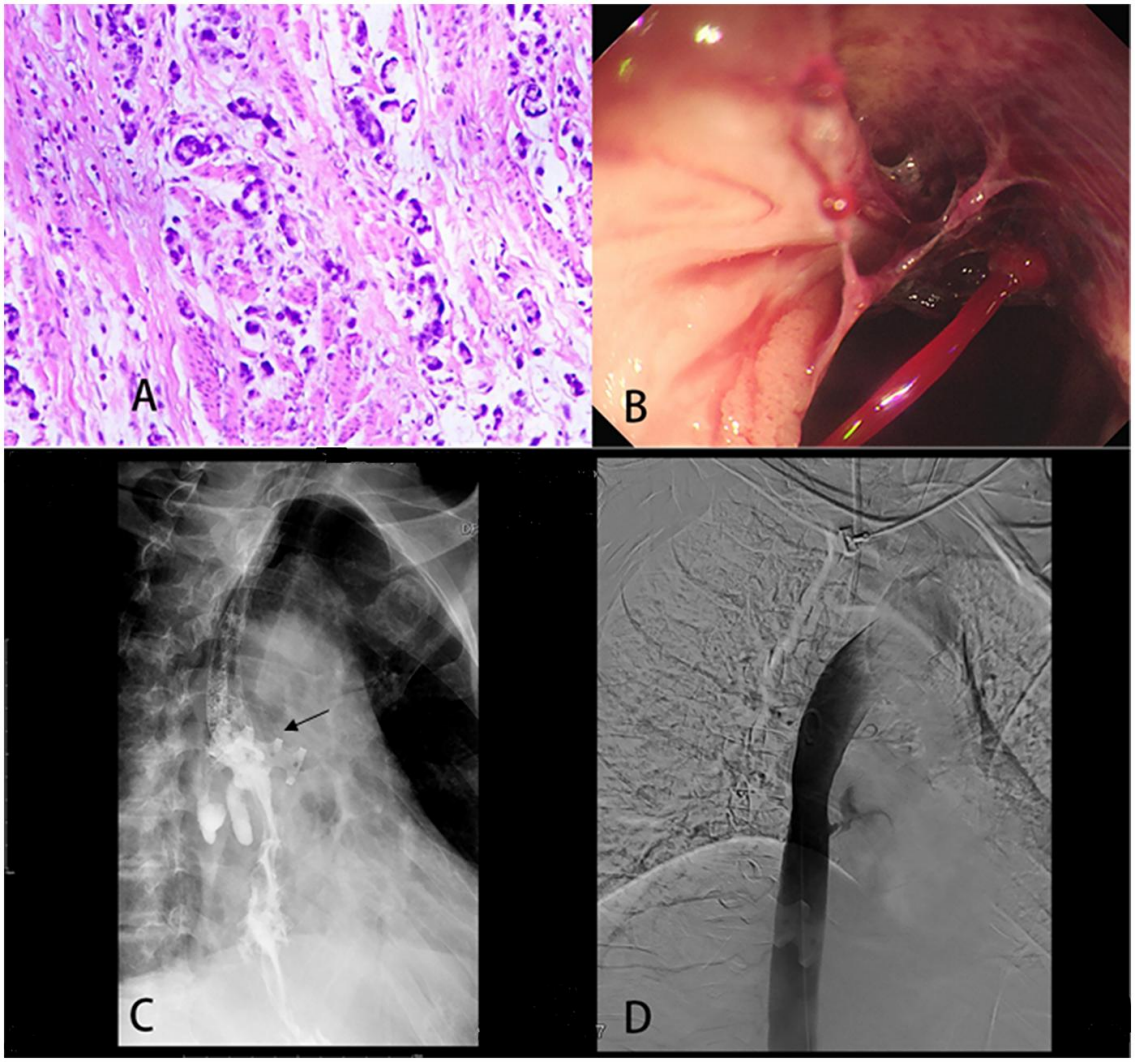
Histopathological, endoscopic, and radiographic features of aortoesophageal fistula following surgical resection for gastric cardia carcinoma. **(A)** Immuno histochemical staining of the resected gastric cardia specimen confirming signet-ring cell adenocarcinoma. **(B)** Emergency gastroscopy demonstrating active hemorrhage originating from a disrupted vessel at the anastomotic site. **(C)** Upper gastrointestinal barium study delineating the anatomical location of the anastomotic stoma. The location indicated by the arrow is an anastomotic leak. **(D)** Aortographic image revealing contrast extravasation from the fistulous communication.

A 61-year-old male patient underwent left thoracic cardia cancer resection with subaortic esophagogastric anastomosis. The initial restoration of gastrointestinal continuity was achieved through intrathoracic esophagogastric anastomosis. The scope of lymph node dissection encompassed both the abdominal cavity and thoracic cavity (lower mediastinum), centering on D2 lymphadenectomy. Abdominal regional lymph nodes (key areas for dissection): Left gastric artery area: includes groups 1, 3, and 7 lymph nodes. Group 1: right cardial lymph nodes. Group 3: lesser curvature lymph nodes. Group 7: left gastric artery trunk lymph nodes. This is one of the most common lymph node groups for metastasis in cardia cancer and must be thoroughly dissected. Celiac trunk area: includes group 9 lymph nodes. Group 9: lymph nodes around the celiac trunk. These are important second-station lymph nodes. Splenic artery area: includes groups 10 and 11 lymph nodes. Group 10: splenic hilar lymph nodes (important for upper gastric cancer, especially near the greater curvature). Group 11: splenic artery trunk lymph nodes. Common hepatic artery area: includes group 8 lymph nodes. Group 8: common hepatic artery trunk lymph nodes. Other related groups: depending on tumor location and extent of invasion, groups 2 (left cardial lymph nodes) and 4 (greater curvature lymph nodes) may also need to be dissected. II. Thoracic regional lymph nodes (accessible via left thoracic approach) Through the left thoracic incision, lower mediastinal lymph nodes can be dissected. This dissection mainly included Group 110 (paraeophageal lymph nodes) and Group 111 (supradiaphragmatic lymph nodes). Postoperative pathological analysis confirmed a mucinous adenocarcinoma with signet-ring cell components (Figure 1A), accompanied by tumor infiltration into the omental tissue. On the sixth postoperative day, an anastomotic leak was identified via an Iohexol contrast study, prompting the percutaneous placement of a nasojejunal feeding tube for nutritional support.

### Clinical findings

About one month after the first surgery, experienced sudden hematemesis, expelling approximately 500 mL of blood mixed with gastric contents. Simultaneously, fresh blood was noted in the chest drainage tube. Emergency acid-suppressive treatment was initiated immediately. An urgent digital subtraction angiography (DSA) was performed to evaluate the thoracic aorta, abdominal aorta, and superior mesenteric artery; however, no significant contrast extravasation was observed. During this procedure, a Progreat microcatheter was advanced to the right gastroepiploic artery, which was subsequently embolized as a preventive measure.

### Diagnostic assessment

**Figure 2:**
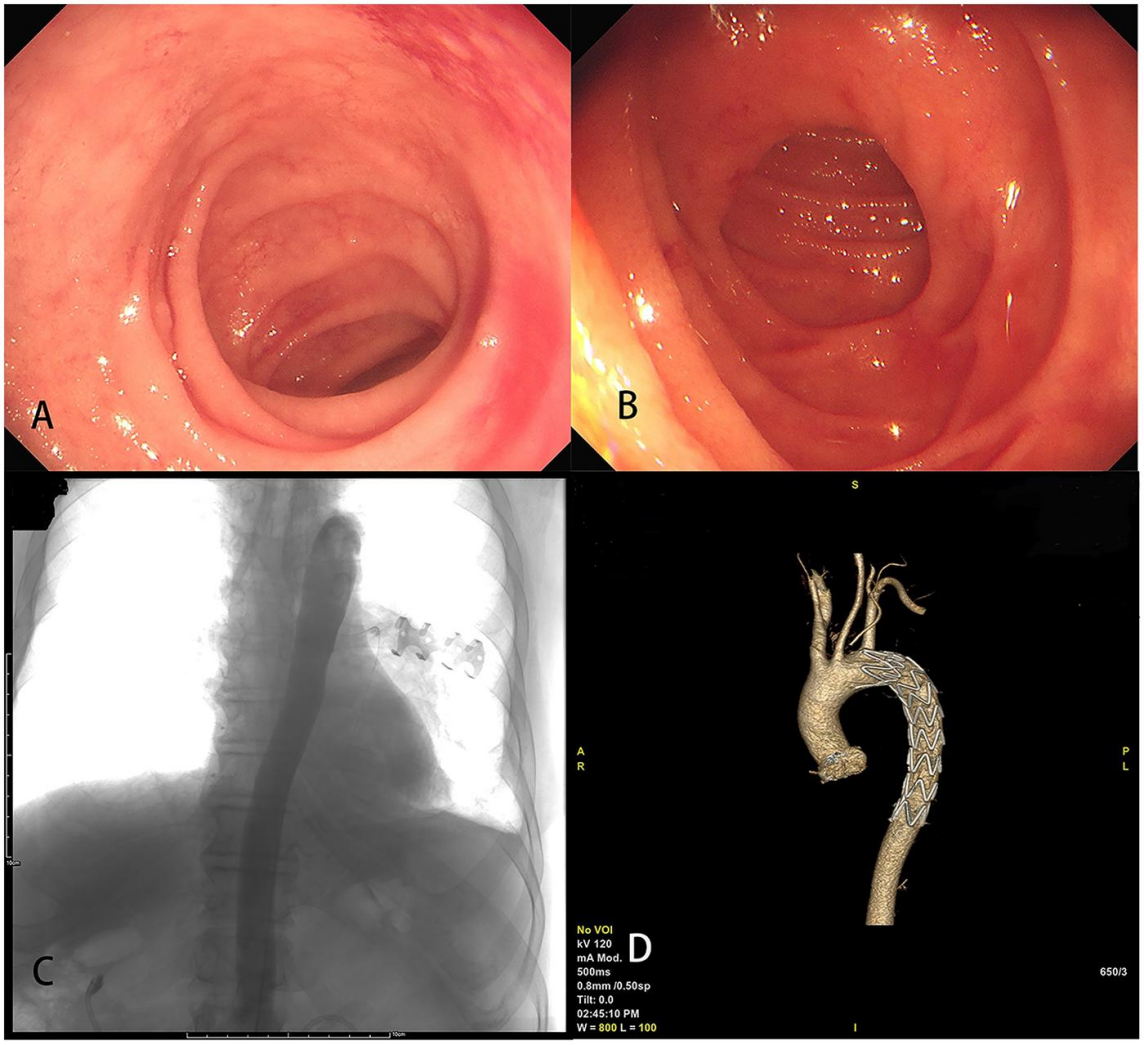
Post-procedural endoscopic and imaging assessment after multidisciplinary management of aortoesophageal fistula. **(A)** Endoscopic examination at one-month follow-up reveals well-healed esophageal mucosa with no signs of active bleeding or fistula recurrence. **(B)** Surveillance endoscopy performed six months postoperatively demonstrates sustained mucosal healing without evidence of luminal stenosis or recurrent fistula. **(C)** Thoracic aortography six months after intervention confirms successful exclusion of the fistula, with no contrast extravasation, indicating complete vascular sealing. **(D)** Postoperative thoracic computed tomography angiography (CTA) acquired one week following stent-graft deployment shows appropriate positioning and fixation of the endovascular prosthesis, with no endoleak or residual hemorrhage.

Following the DSA, the patient experienced recurrent hematemesis, prompting an initial upper gastrointestinal contrast study followed by gastroscopic evaluation. The patient's blood pressure plummeted to 65/40 mmHg, requiring emergency chest surgery. Intraoperatively, multiple internal adhesions were identified; following lysis, a large dilated segment of the stomach containing dark fluid was observed within the thoracic cavity. Upon opening this segment, bright red gastric contents were released under pressure, and a pulsating jet of blood was observed emanating from a 0.7 cm defect adjacent to the surgical anastomosis site, indicating an anastomotic-arterial fistula. Prior emergency gastroscopy had revealed active bleeding from the anastomosis (Figure 1B), and the upper GI series had demonstrated the location of the anastomotic leak (Figure 1C). Subsequent aortography clearly showed the site of contrast extravasation from the fistula (Figure 1D). Figure 2 summarizes the key timeline for this clinical event.

### Therapeutic intervention

The management of this complex case was indeed a collaborative effort from the outset. A multidisciplinary team (MDT) involving cardiothoracic surgery, gastrointestinal surgery, vascular surgery, interventional radiology, and intensive care was convened for all major decisions. During the operation, efforts were made to repair the aortic wall defect primarily, but direct suturing failed due to severe tissue swelling and weakness. The patient experienced recurrent episodes of profound hypotension and life-threatening arrhythmias, necessitating aggressive hemorrhage control and vasopressor support. Subsequently, a stent-graft was placed in the thoracic aorta via the femoral artery to exclude the fistula. The procedure was performed under general anesthesia. Total procedural time was 80 min. Vascular access was obtained via percutaneous femoral artery puncture. Under fluoroscopic guidance, a guidewire was advanced, followed by a catheter, to navigate to the target aortic segment. Diagnostic angiography was first performed to confirm the precise location and morphology of the aortoesophageal fistula. Subsequently, a suitably sized endovascular stent-graft was selected based on preoperative imaging measurements. The stent-graft delivery system was advanced over the guidewire, precisely positioned across the fistula site, and deployed. Immediate completion angiography confirmed successful exclusion of the fistula with no evidence of endo leak. The patient received a massive blood transfusion and treatment for shock, with vital signs slowly improving. The definitive surgical treatment ultimately involved closure and burial of the intrathoracic gastric conduit segment, esophageal exclusion, and a jejunostomy. The patient required a prolonged period of nothing-by-mouth (NPO) to allow for healing of the thoracic cavity and to prepare for the definitive colon interposition. The jejunostomy provided reliable, long-term enteral access for full nutritional support, which is critical for optimizing the patient's nutritional status, wound healing, and immune function before and after the major reconstructive surgery. Postoperatively, the patient's vital signs remained stable.

The management of aortoesophageal fistula (AEF) has evolved from a singular, high-risk surgical approach into a nuanced, multi-modal strategy. Recognizing that patient safety must be balanced with infection control, clinicians now tailor care using a spectrum of options ranging from open repair and endovascular techniques to hybrid methods and palliative support. Thoracic endovascular aortic repair (TEVAR) serves as a critical bridge: it rapidly arrests hemorrhage and stabilizes the patient, acting as a temporary shield against immediate mortality. However, because stent grafts remain susceptible to secondary infection, TEVAR is rarely a definitive cure; rather, it provides a temporal bridge for subsequent interventions to address sepsis and esophageal integrity. While traditional open repair offers the potential for complete eradication of infection, its formidable physiological toll restricts its use to only the most robust candidates. Alternative surgical strategies, such as *in-situ* graft replacement with omental wrapping or extra-anatomic bypass, provide superior long-term infection control compared to primary stenting, albeit with heightened perioperative risks. Ultimately, for patients too frail to withstand any surgical trauma, conservative or palliative measures remain indispensable, offering symptom relief even though they cannot resolve the underlying pathology.

### Follow-up and outcomes

In the early postoperative period, the patient experienced occasional fever which was managed with antibiotics. Mechanical ventilation was discontinued. Blood counts remained stable without evidence of new bleeding, and inflammatory markers did not show a significant increase. The patient was discharged upon achieving stability. Five months later, a second operation was performed using laparoscopic keyhole surgery to close the esophagus and reconstruct the alimentary tract with a segment of colon. The colon was anastomosed to the upper esophagus in the neck behind the sternum. Following this procedure, the patient tolerated oral intake well, reporting only occasional dysphagia and reflux.

The case was presented to the multidisciplinary oncology tumor board following the patient's recovery from reconstructive surgery. The initial cardiac adenocarcinoma was treated with curative intent via proximal gastrectomy to achieve R0 resection. Subsequent life-threatening complications such as sepsis and AEF combined with extensive salvage surgeries changed the risk-benefit ratio for adjuvant chemotherapy. A prolonged recovery period combined with a compromised nutritional state and the physiological stress of multiple major operations increased the risks of chemotherapy-related toxicity. The multidisciplinary oncology team and the patient made a shared decision to forgo adjuvant chemotherapy and adopt a strategy of close surveillance.

**Figure 3:**
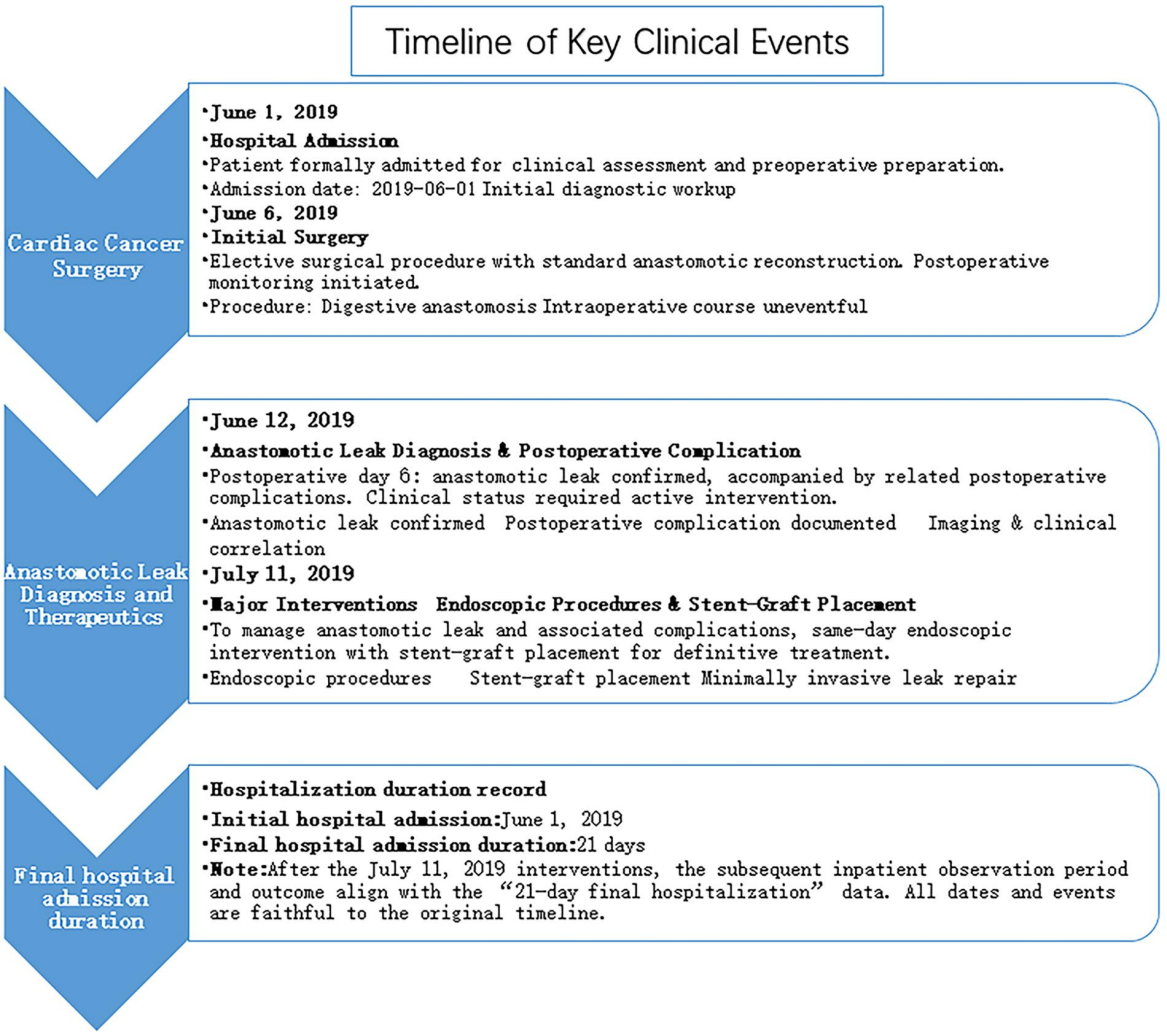
Timeline of key clinical events from June 1 to July 11, 2019. The patient was admitted for preoperative preparation (June 1), underwent initial digestive anastomosis surgery (June 6), developed a confirmed anastomotic leak on postoperative day 6 (June 12), and received endoscopic stent-graft placement for leak management (July 11). The total initial hospitalization duration was 21 days.

Follow-up endoscopy at one month showed well-healed esophageal mucosa with no bleeding or fistula recurrence (Figure 3A). Examination at six months confirmed continued healing without narrowing (Figure 3B). Aortography six months after surgery confirmed successful closure of the fistula with no leakage (Figure 3C). A postoperative CT angiogram one week after stent placement showed the stent was in a good position with no signs of leakage (Figure 3D). At a follow-up visit three years later, the patient was alive and had not experienced hematemesis again. Follow-up assessments indicated significant symptomatic improvement after hemorrhage cessation, with the patient demonstrating an understanding of the necessity for regular monitoring and adherence to the treatment regimen.

## Discussion

The formation of an aortoesophageal fistula after radical gastrectomy for gastric cardia cancer is a exceedingly rare and severe complication that typically progresses through anastomotic leakage, mediastinal infection, and subsequent aortic erosion (7). Although this progression is described in the literature, the diagnostic presentation in our specific case was markedly atypical. The characteristic Chiari triad of chest pain, sentinel hemorrhage, and exsanguination was not fully manifest, and the initial digital subtraction angiography (DSA) yielded a false-negative result for active extravasation (8). This diagnostic dilemma highlights a critical challenge because a single negative test can dangerously delay treatment when clinical suspicion is high. Relying on one method alone is not insufficient, as evidenced by other reports of AEF with subtle early signs (9). The initial false-negative DSA in our patient, potentially attributable to intermittent fistula patency or thrombus seal, highlights a key limitation of this imaging method and underscores the necessity for serial clinical and imaging assessments in high-risk postoperative cases (10). Endoscopic stenting for a contained anastomotic leak was considered. However, it was deemed unsuitable in this specific scenario because the leak had already progressed to form a direct fistula with the thoracic aorta. Placing a stent across a fistula communicating with a major artery carries a high risk of precipitating massive hemorrhage and would not address the aortic wall defect. In therapeutic management, the emergency use of thoracic endovascular aortic repair (TEVAR) for immediate hemostasis in our case aligns with current literature supporting this endovascular method as a vital life-saving measure to stabilize hemodynamics (11, 12). Our management strategy differs by using a planned two-stage surgery. Unlike cases where TEVAR alone is the final or palliative treatment, our patient had esophageal exclusion followed by delayed colonic interposition (13). This approach addresses both the residual infection site and the critical necessity for permanent digestive tract reconstruction. These factors are crucial for long-term survival yet are frequently under discussed in cases involving fistulas following gastrectomy (14). The successful long-term outcome in our patient, with survival exceeding three years, contrasts with the historically dismal prognosis and supports the efficacy of a structured, hybrid multidisciplinary strategy that integrates endovascular salvage with definitive surgical source control and reconstruction (15). This case further illustrates key teaching points about diagnostic challenges, underlying disease processes, and complex treatment decisions. The initial false-negative digital subtraction angiography (DSA), despite strong clinical suspicion, represents a significant diagnostic pitfall. This can happen due to intermittent fistula flow, temporary clot blockage, or pressure from nearby structures, meaning a single negative angiogram cannot rule out AEF when clinical risk is high (16). Clinicians must maintain a high index of suspicion because early symptoms such as non-specific chest pain or minor hematemesis can be misleadingly attributed to more common postoperative conditions and lead to dangerous delays (17). The patient's disease progression shows a distinct pattern starting from surgical injury and anastomotic failure led to persistent leakage and local infection, subsequently causing enzymatic and inflammatory damage to the adjacent aortic wall and resultant fistula formation (18). This highlights the critical importance of postoperative mediastinal infection as the main cause of vascular complications. Management of such severe cases requires careful strategic considerations.

While emergency TEVAR is crucial for immediate bleeding control, it serves primarily as a temporizing measure rather than a definitive solution, as it does not address the underlying infection or esophageal tear, which can lead to ongoing sepsis, endo leak, or graft damage (19). This case underscores the necessity of a coordinated, multidisciplinary approach. After life-saving TEVAR, proper source control with esophageal exclusion and diversion is key to handle the septic focus and stabilize the mediastinum (20). Performing a planned colonic interposition later is essential for rebuilding the digestive tract, restoring its function, and ensuring lasting results (21). The definitive surgery (colon interposition) was performed for several key reasons: (1) Complete resection of the previously infected and necrotic gastric conduit and the fistula tract was required to eliminate the ongoing source of infection and prevent recurrence. (2) After resection of the compromised stomach, a new, viable conduit was required to re-establish gastrointestinal continuity. The left colon, supplied by the ascending branch of the left colic artery, was chosen due to its reliable vascular pedicle, sufficient length to reach the neck without tension, and its location outside the previous infected field. (3) The new cervical esophago-colonic anastomosis was created in a clean, non-irradiated field, significantly reducing the risk of another anastomotic complication. This staged hybrid strategy combines endovascular general thoracic and reconstructive principles, demonstrating that a carefully planned series of interventions can provide significant survival benefit even for a complication historically linked to near-universal mortality-a finding supported by our patient's three-year follow-up (22).

Conventional open surgical management of AEF, involving graft excision, aortic stump closure, and bowel repair—often with extra-anatomic bypass—is associated with exceptionally high morbidity and mortality rates. The procedures are physiologically demanding, and patients frequently present in extremis, making them unsuitable for such major vascular surgery. Perioperative mortality remains daunting, and even when attempted, outcomes can be poor, especially in unstable patients or resource-limited settings (23). Endovascular aortic repair (EVAR) techniques have emerged as a vital tool for the urgent control of life-threatening hemorrhage from an AEF. The placement of an endovascular stent graft can effectively serve as temporizing measure by sealing the fistula and stabilizing the hemodynamically unstable patient (22). This approach is less invasive and can be a bridge to definitive therapy, allowing for patient optimization. In emergency contexts, even physician-modified endo grafts may be utilized when standard devices are anatomically unsuitable (24). Contemporary management of AEF emphasizes a multidisciplinary team approach and a staged strategy. The initial goal is to control acute bleeding, typically with endovascular means, and manage sepsis with broad-spectrum antibiotics. Once stabilized, the patient can undergo a planned, definitive open repair. This two-stage approach—endovascular temporization followed by open reconstruction—aims to improve outcomes by avoiding high-risk surgery in an unstable patient (25). The management of aortoenteric fistula (AEF) presenting with massive gastrointestinal bleeding necessitates immediate and aggressive hemodynamic resuscitation, often guided by a massive transfusion protocol (MTP). Patients frequently present with hemorrhagic shock, requiring rapid volume replacement and blood product administration to restore circulating volume and oxygen-carrying capacity. The critical nature of this bleeding is underscored by cases where patients experience sudden episodes of hematemesis or melena leading to profound shock, demanding emergent intervention (26). The high mortality associated with AEF is partly due to the challenge of achieving hemostasis in the setting of such catastrophic blood loss, as seen in cases where patients succumbed preoperatively or within 24 h despite surgical efforts (27). Early recognition of hemodynamic instability is paramount, as these patients often require vasopressor support and have a significantly higher mortality compared to other causes of post-aortic surgery bleeding (28). The resection of the involved intestinal segment is a cornerstone of definitive source control in aortoenteric fistula (AEF) management, as the fistula represents a persistent source of sepsis and life-threatening hemorrhage. The necessity for resection is absolute, given the high mortality associated with untreated AEFs (29). The scope of resection must be carefully defined to ensure complete removal of all infected and necrotic tissue, which is critical for preventing recurrence. This typically involves resection of the fistulized bowel segment, most commonly the third or fourth portion of the duodenum (30). In complex cases, such as double secondary AEFs, the resection may need to extend to multiple sites, including unusual locations like the cecum, underscoring the importance of thorough intraoperative assessment to define the full anatomical extent of the disease (30). This case highlights the need to suspect an aortoenteric fistula in any post-gastrectomy patient with an anastomotic leak who has hematemesis, even if initial imaging is negative. Aggressive diagnostic methods such as the combination of CT angiography with endoscopy are recommended, and hospitals should establish rapid response plans for major postoperative bleeding. The successful outcome here demonstrates that a staged, multidisciplinary approach using TEVAR, surgery, and reconstruction be effective, although this represents a single-case observation. Timely detection and specialized care likely contributed to this favorable outcome. Future studies should identify risk factors, early diagnostic signs, and better management strategies for this serious complication.

## Data Availability

The data supporting this case report are available from the corresponding author upon reasonable request.
